# Summarizing the 2024 immunotherapy manual of the Canadian society of allergy and clinical immunology

**DOI:** 10.1186/s13223-026-01020-6

**Published:** 2026-03-09

**Authors:** Aria Bruton Joe, George Cai, Samina Nazarali, Harold Kim, Godfrey Lam, Susan Waserman

**Affiliations:** 1 Department of Medicine, Division of Allergy and Immunology, University of British Columbia, Vancouver, BC Canada; 2 Department of Medicine, Division of Clinical Immunology and Allergy, McMaster University, Hamilton, ON Canada; 3 Department of Medicine, Division of Clinical Immunology and Allergy, Western University, London, ON Canada

**Keywords:** Allergen immunotherapy, Subcutaneous immunotherapy, Sublingual immunotherapy, Venom immunotherapy, Immunotherapy manual, Immunotherapy

## Abstract

Allergen immunotherapy (AIT) is an essential disease-modifying therapy in the field of allergy and clinical immunology. The Immunotherapy Manual of the Canadian Society of Allergy and Clinical Immunology serves as a comprehensive guide for the safe and effective administration of immunotherapy. Its purpose is to address an educational gap in immunotherapy identified by residency training programs in Canada.

This summary highlights key updates for both aeroallergen and venom immunotherapy from the latest edition of the immunotherapy manual and provides a concise, accessible resource for trainees to support their practice. The areas reviewed include principles of immunotherapy, allergen extract standardization, and safety related to immunotherapy with practical strategies for risk mitigation. Included in the original manual are practice cases that have been updated with systematic checklists to coach readers in their management of patients.

The updated immunotherapy manual and its summary aim to serve as a basis for trainees as they develop competence in the provision of immunotherapy.

## Background

The immunotherapy manual of the Canadian Society of Allergy and Clinical Immunology (CSACI) has been instrumental in supporting current and future clinicians who practice immunotherapy [1]. Drs. Kim, Lam and Waserman, supported by their collaborators, have recently updated the third edition of this important educational resource. Allergen immunotherapy (AIT) has been utilized for over a century in our field for the treatment of allergic conditions that are not adequately managed by allergen avoidance and optimal medical therapy or based on patient preference [2]. Unlike other treatments for allergic conditions, both subcutaneous immunotherapy (SCIT) and more recently, sublingual immunotherapy (SLIT) have shown disease-modification [3].

This summary provides an overview of the 2024 Immunotherapy Manual and highlights changes from the 2016 version. The Immunotherapy Manual, this summary, and the immunotherapy pocket guide serve as foundational resources to support trainees as they develop their own clinical expertise and can be downloaded through this link: https://www.csaci.ca/wp-content/uploads/2024/12/IT-manual-Dec-13_2024_CLEAN.pdf.

### What’s new and what’s different

- There is one fewer standardized cat extract available compared with 2016.
- A new, more potent dog extract (acetone-precipitated dog) has been introduced.
- There is inconsistent availability of pre-seasonal SCIT.
- Dosing recommendations for SCIT have changed, in particular, non-standardized allergens should be dosed based on protein nitrogen units (PNU) corresponding to a 1:200 w/v concentration.
- Beta blocker use is now considered a relative, rather than absolute, contraindication to immunotherapy.
- Indications for SLIT have expanded, with all SLIT tablets now approved for use in patients as young as five years of age.
- New recommendations address practical aspects of SCIT administration, including dose adjustments following missed doses and following systemic reactions.
- Evidence now supports premedication with antihistamines prior to SCIT administration.

### Fundamentals of allergen immunotherapy

AIT is effective in the management of allergic rhinitis, allergic asthma, atopic dermatitis, and stinging insect hypersensitivity (Table 1). It is a disease-modifying therapy for appropriate candidates sensitized to the relevant allergens. Inhalant allergen immunotherapy is recommended for patients in whom pharmacotherapy has failed or caused adverse events. Patients may also prefer to avoid pharmacotherapy and pursue a disease-modifying treatment approach [3]. VIT is indicated in patients with a history of anaphylaxis to stinging insects and can also be considered in those with large local reactions or cutaneous systemic reactions [4]. A thorough evaluation of clinical and medication history, and appropriate skin and or blood testing, are critical for selecting the appropriate patients and allergens for AIT.

**Table 1:** Allergens for which there is evidence-based efficacy for the treatment of allergic conditions

Aeroallergen	Allergic rhinitis/ conjunctivitis	Asthma	Atopic dermatitis	Stinging insect hyper- sensitivity
birch pollen	x			
mountain cedar pollen	x			
grass pollen	x	x		
ragweed pollen	x	x		
Russian thistle pollen	x			
Parietaria	x			
house dust mite	x	x	x	
cockroach	x			
cat	x	x		
dog	x	x		
Alternaria	x	x		
Cladosporium herbarum	x	x		
Hymenoptera				x

### Perennial subcutaneous immunotherapy

SCIT involves gradually introducing increasing doses of clinically relevant allergens until a therapeutic maintenance dose is reached to induce tolerance. For most patients, it is recommended that each SCIT vial contains a maximum of three to four allergens. Note that some allergen extracts, such as Alternaria and cockroach, contain proteolytic enzymes and should be prescribed in separate vials [5, 6]. Tree pollens also pose a challenge, as many have limited cross-reactivity with each other and need to be individually represented (Table 2) [7]. Prescribers should use a mix of tree pollen extracts that are representative of the patient’s geographic area, and where there is evidence of sensitization.

SCIT is administered with allergen extracts which can be standardized or non-standardized. The standardization of allergen extracts aims to achieve consistency in their content and potency [8]. In North America, this involves methods such as ID50EAL, which measures the intradermal skin test sizes of different dilutions in sensitized subjects. Extracts producing similar skin test responses are considered bioequivalent. In Canada, there are 18 standardized extracts which include hymenoptera venoms (6), grass pollens (8), cat (1), ragweed (1), and house dust mites (2). Of note, there is one fewer standardized cat extract than in 2016. SCIT can still be safely and effectively administered with non-standardized extracts. For non-standardized extracts, the manual suggests a final dose in protein nitrogen units (PNU) that corresponds to a 1:200 w/v. This was achieved by surveying Canadian allergen extract manufacturers for historical PNU values that reached 1:200 w/v, and taking the average PNU numbers across different manufacturers. *This differs from the 2016 manual*,* which advises the use of 5000 PNU/mL for non-standardized extracts. The change reflects a more consistent approach*,* given the variability in concentration across non-standardized allergen extracts corresponding to 5000 PNU/mL* [9].

**Table 2:** Relevant tree pollen cross-reactivity

Aeroallergen
Cottonwood, poplar, willow and aspen pollens
Oak, beech, chestnut, birch, hazel, and alder pollens
Ash, European olive, Russian olive, and privet pollens
Juniper, cedar, cypress, and *arborvitae* pollens
Walnut and hickory pollens

The updated manual contains a dosing schedule with key changes from 2016. These include dosing changes based on 1/200 w/v, for non-standardized allergens such as birch pollen, Alternaria, acetone precipitated (AP) and ultrafiltered (UF) dog (Table 3). The recommended PNU dosing for non-standardized allergens in the manual reflects an approximation of a safe and effective dose that can be applied across different manufacturers. It is advised that prescribers consult the specific manufacturer for the most current PNU dose which reflects the 1:200 w/v.

**Table 3:** Recommended therapeutic allergen dose/ml, presuming 0.5 ml/maintenance dose

Allergen	CSACI 2024 recommended dose/ml
*D. pteronyssinus*	2000 AU
*D. farinae*	2000 AU
Dust mite mix	2000 AU mix
Cat	2000 BAU
Dog*	30 mcg
Grass pollen	5000 BAU
Ragweed pollen	5000 PNU
Birch pollen*	2500 PNU
Alternaria*	1000 PNU

*Recommended dose changes from 2016 manual

For the preparation of immunotherapy treatment sets, serial dilutions of allergen extracts are created from full-strength vials, with patients starting at the weakest dilution and escalating on a weekly basis until the monthly maintenance dose is reached. It is recommended that treatment sets of four dilutions are used, although some practitioners will use sets of three dilutions, and there are also rush and cluster protocols available. Rush and cluster protocols are associated with significantly increased risk of systemic reactions and thus not commonly used in most centers.

With SCIT, it is imperative to counsel patients on the risk of systemic reactions. It is estimated that systemic reactions occur in up to 7% of patients undergoing SCIT. If SCIT continues following a systemic reaction, the subsequent dose should be reduced to 10% of the previous dose (for severe anaphylaxis) or 50% of the previous dose (for milder systemic reactions). It is uncertain whether large local reactions during immunotherapy increase the risk of systemic reactions; therefore, most allergists are likely to repeat the dose or build up more gradually [10, 11]. Additionally, prescribers can consider the use of antihistamines and montelukast, which may reduce the risk of local reactions [2].

### Pre-seasonal SCIT

Pre-seasonal SCIT is an alternative to conventional SCIT where injections are given year-round. In pre-seasonal SCIT, therapeutic benefit is derived from injections completed before the season. Once the maintenance dose is reached, the benefit is sustained for the duration of the pollen season. There is a scarcity of data supporting the efficacy and safety of pre-seasonal SCIT; however, its clinical benefit has been reported [12]. Currently, pre-seasonal SCIT is available for tree, grass, and ragweed pollens. There is some uncertainty as to whether these alum-precipitated extracts will always be reliably available ongoing.

### Sublingual immunotherapy

SLIT is another method of desensitizing patients by administering relevant allergen extract tablets sublingually to induce tolerance. In Canada, it is currently available for the treatment of allergies to house dust mite, as well as birch, grass and ragweed pollen. SLIT offers several advantages over SCIT, including fewer systemic reactions, and the ability to administer treatment at home after the first dose has been given under medical supervision [13]. In addition, tablets for grass, ragweed and birch pollen can be initiated prior to the season and do not need to be taken year-round (Table 4). SLIT is generally delivered for three to five years. Common side effects of SLIT include oropharyngeal pruritus, discomfort, and swelling [14, 15]. There is a very small risk of more severe systemic allergic reactions [15]. While limited to case reports, an association has been noted between eosinophilic esophagitis and aeroallergen SLIT [16, 17].

**Table 4:** SLIT products and dosing

Product	Dose	When to start (first tablet given in the office setting, subsequent doses at home)	When to stop
Oralair ^®^ (grass pollen SLIT)	First: 100 IR Second: 200 IR Subsequent: 300 IR	16 weeks before grass pollen season Note: only the first dose (100 IR) needs to be in office.	End of grass pollen season*
Grastek ^®^ (grass pollen SLIT)	2800 BAU	8–12 weeks before grass pollen season	End of grass pollen season*
Ragwitek ^®^ (ragweed pollen SLIT)	12 amb a 1-U	12 weeks before ragweed pollen season	End of ragweed pollen season*
Acarizax ^®^ (dust mite SLIT)	12 SQ-HDM	Anytime	Continue for 3–5 years
Itulatek ^®^ (birch pollen SLIT)	12 SQ-Bet	16 weeks before birch pollen season	End of birch pollen season*

*These products can be administered perennially. Based on available evidence, they do not seem to be more effective than co-seasonal administration after the first pollen season

### Venom immunotherapy

Venom from stinging insects can be associated with a range of reactions, from limited local reactions to anaphylaxis. Between 0.4 and 0.8% of children and up to 3% of adults will experience systemic reactions to Hymenoptera stings [4, 18]. Although patients with large local reactions or severe reactions limited to the skin are not at significantly increased risk of anaphylaxis, patients who experience anaphylaxis are at high risk of recurrence [4]. In patients with life-threatening reactions, VIT can reduce the risk of anaphylaxis to < 5% with subsequent stings, making it a significant and life-saving intervention [19, 20]. VIT can also be considered for bothersome large-local reactions or cutaneous systemic reactions after shared decision-making with the patient.

Common culprits include yellow jacket, hornets (yellow and white-faced), wasp and honeybee. Hornets and yellow jacket are noted to significantly cross-react, meaning that VIT to one species protects against the others; however, this cross-reactivity decreases with other species such as wasp, and honeybee is the least cross-reactive with other species [4]. The investigation of patients for venom allergy involves skin testing and/or serum-specific IgE testing to the individual venoms.

VIT protocols vary among allergists, but many centers use a modified rush protocol to build patients up to a maintenance dose within 6–8 weeks [21]. Maintenance doses are given every four to twelve weeks, depending on the duration of immunotherapy, and are continued for three to five years. Select patients may choose to continue indefinitely, particularly those with very severe reactions, sensitization to honeybee, mast cell disorders or a high frequency of exposure (e.g. occupational).

### Practical safety issues

Allergen immunotherapy carries the risk of severe and potentially fatal anaphylaxis [22]. Physicians must be aware of contraindications to immunotherapy and recognize patient-specific risk factors for anaphylaxis. Immunotherapy is contraindicated in individuals with poorly controlled or severe asthma and relatively contraindicated in those with significant comorbid conditions, such as cardiovascular or respiratory disease [23]. Key risk factors for anaphylaxis include asthma that is not optimally controlled, asthma symptoms present immediately before immunotherapy administration, a history of systemic reactions to allergen immunotherapy, and receiving treatment from a new maintenance vial [23]. Additionally, concurrent treatment with beta-blockers or ACE-inhibitors may increase the severity of reactions to inhalant SCIT [24], and potentially interfere with efficacy of epinephrine administration. The same risk has not been observed with VIT nor SLIT [14, 25].

To minimize risks, physicians should take appropriate precautions, including avoiding dosing errors and ensuring that SCIT injections are not administered intravascularly or intramuscularly. Patients must be informed about the risks of immunotherapy and educated on the recognition of anaphylaxis and on strategies to reduce those risks (Table 5).

**Table 5:** Comparing the risks and safety considerations of subcutaneous immunotherapy (SCIT) and Sublingual immunotherapy (SLIT)

Risk of anaphylaxis	SCIT	SLIT
	1–12.7% [23]	< 1% [15]
Risk of localized complications	35% risk of localized swelling at the site of injection [10]	40% risk of oral itching, throat and ear discomfort, and oral swelling [14] Risk of eosinophilic esophagitis [16, 17]
Safety considerations	30 min observation period post-injection Avoid exercising 2 h post-injection Avoid in the setting of fever, respiratory infection, or increased allergy symptoms	Skip a dose if an open wound is present in the oral mucosa

It is imperative that healthcare providers administering immunotherapy are well-equipped and competent at managing anaphylaxis. Clinics offering immunotherapy should maintain a checklist of essential supplies for anaphylaxis management (Table 6). After an episode of anaphylaxis, the physician should attempt to identify its cause, adjust immunotherapy where appropriate, and engage in shared decision making with their patient on the course of immunotherapy going forward [24].

**Table 6:** Checklist with mandatory supplies for the management of anaphylaxis

Supplies	Available (x)
epinephrine 1:1000 (most important)	
oral antihistamines	
injectable antihistamines	
salbutamol or comparable fast-acting bronchodilator	
glucagon	
tourniquets	
IV access	
IV tubing for fluids	
normal saline or Ringer’s lactate	
oxygen	
Ambu bag	
oropharyngeal airway	

## Conclusions

The updated immunotherapy manual of the Canadian Society of Allergy and Clinical Immunology aims to overcome an educational gap in allergen immunotherapy. By summarizing key principles, practical considerations, and safety parameters, this document can help trainees gain confidence and competence in the provision of immunotherapy. As allergen immunotherapy is the only immunomodulatory treatment for allergic diseases, acquisition of expertise and ongoing education in this area are essential for the optimization of patient outcomes.

## Data Availability

No datasets were generated or analysed during the current study.

## Abbreviations

AIT: Allergen immunotherapy
AP: Acetone precipitated
CSACI: Canadian society of allergy and clinical immunology
PNU: Protein nitrogen units
SCIT: Subcutaneous immunotherapy
SLIT: Sublingual immunotherapy
UF: Ultrafiltered
VIT: Venom immunotherapy

## References

[1] Kim H, Lam G, Waserman S. 2024 Immunotherapy Manual. Canadian Society of Allergy & Clinical Immunology. 2024. Accessed 31 Dec 2024. https://www.csaci.ca/wp-content/uploads/2024/12/IT-manual-Dec-13_2024_CLEAN.pdf 10.1186/s13223-026-01020-6 PMC13085749 41803996

[2] Calderon MA, Waserman S, Bernstein DI, et al. Clinical practice of allergy immunotherapy for allergic rhinoconjunctivitis and asthma: an expert panel report. J Allergy Clin Immunol Pract. 2020;8(9):2920–e361. 32422372 10.1016/j.jaip.2020.04.071

[3] Roxbury CR, Lin SY. Efficacy and safety of subcutaneous and Sublingual immunotherapy for allergic rhinoconjunctivitis and asthma. Otolaryngol Clin N Am. 2017;50(6):1111–9. 10.1016/j.otc.2017.08.011 28964530

[4] Golden DBK, Demain J, Freeman T, Graft D, Tankersley M, Tracy J, et al. Stinging insect hypersensitivity. Ann Allergy Asthma Immunol. 2017;118(1):28–54. 28007086 10.1016/j.anai.2016.10.031

[5] Esch RE. Allergen immunotherapy: what can and cannot be mixed? J Allergy Clin Immunol. 2008;122(3):659–60. 18774400 10.1016/j.jaci.2008.07.018

[6] Grier TJ, LeFevre DM, Duncan EA, Esch RE, Coyne TC. Allergen stabilities and compatibilities in mixtures of high-protease fungal and insect extracts. Ann Allergy Asthma Immunol. 2012;108(6):439–47. 10.1016/j.anai.2012.04.012 22626598

[7] Weber RW. Patterns of pollen cross-allergenicity. J Allergy Clin Immunol. 2003;112(2):229–39. 12897724 10.1067/mai.2003.1683

[8] Slater JE, Pastor RW. The determination of equivalent doses of standardized allergen vaccines. J Allergy Clin Immunol. 2000;105(3):468–74. 10719295 10.1067/mai.2000.104551

[9] Dua B, Park J, Kim H. Comparison of weight per volume and protein nitrogen units in non-standardized allergen extracts: implications for prescribing subcutaneous immunotherapy. Allergy Asthma Clin Immunol. 2021;17:92. 34530909 10.1186/s13223-021-00588-5 PMC8447704

[10] Roy SR, Sigmon JR, Olivier J, Moffitt JE, Brown DA, Marshall GD. Increased frequency of large local reactions among systemic reactors during subcutaneous allergen immunotherapy. Ann Allergy Asthma Immunol. 2007;99(1):82–6. 17650835 10.1016/S1081-1206(10)60626-6

[11] Calabria CW, Stolfi A, Tankersley MS. The REPEAT study: recognizing and evaluating periodic local reactions in allergen immunotherapy and associated systemic reactions. Ann Allergy Asthma Immunol. 2011;106(1):49–53. 21195945 10.1016/j.anai.2010.10.025

[12] Tworek D, Bochenska-Marciniak M, Kuprys-Lipinska I, Kupczyk M, Kuna P. Perennial is more effective than preseasonal subcutaneous immunotherapy in the treatment of seasonal allergic rhinoconjunctivitis. Am J Rhinol Allergy. 2013;27(4):304–8. 23636036 10.2500/ajra.2013.27.3935 PMC3711679

[13] Nelson HS. Subcutaneous immunotherapy versus Sublingual immunotherapy: which is more effective? J Allergy Clin Immunol Pract. 2014;2(2):144–9. quiz 150–1. 24607040 10.1016/j.jaip.2013.11.018

[14] Canonica GW, Cox L, Pawankar R, Baena-Cagnani CE, Blaiss M, Bonini S, et al. Sublingual immunotherapy: world allergy organization position paper 2013 update. World Allergy Organ J. 2014;7:6. 24679069 10.1186/1939-4551-7-6 PMC3983904

[15] Bernstein DI, Bardelas JA Jr., Svanholm Fogh B, Kaur A, Li Z, Nolte H. A practical guide to the Sublingual immunotherapy tablet adverse event profile: implications for clinical practice. Postgrad Med. 2017;129(6):590–7. 28326906 10.1080/00325481.2017.1302306

[16] Egan M, Atkins D. What is the relationship between eosinophilic esophagitis (EoE) and aeroallergens? Implications for allergen immunotherapy. Curr Allergy Asthma Rep. 2018;18(8):43. 29909507 10.1007/s11882-018-0798-2 PMC8226434

[17] Miehlke S, Alpan O, Schröder S, Straumann A. Induction of eosinophilic esophagitis by Sublingual pollen immunotherapy. Case Rep Gastroenterol. 2013;7(3):363–8. 24163646 10.1159/000355161 PMC3806675

[18] Cichocka-Jarosz E, Brzyski P, Tarczoń I, Jedynak-Wąsowicz U, Tomasik T, Lis G. Quality of life in parents of children and adolescents after systemic Sting reactions. Ann Agric Environ Med. 2019;26(2):315–21. 31232065 10.26444/aaem/93748

[19] Golden DBK, Breisch NL, Hamilton RG, et al. Clinical and entomological factors influence the outcome of sting challenge studies. J Allergy Clin Immunol. 2006;117(3):670–5. 16522469 10.1016/j.jaci.2005.12.1313

[20] Reisman RE. Natural history of insect Sting allergy: relationship of severity of symptoms of initial Sting anaphylaxis to re-sting reactions. J Allergy Clin Immunol. 1992;90(3 Pt 1):335–9. 1345753 10.1016/s0091-6749(05)80012-0

[21] Brown SGA, Wiese MD, van Eeden P, et al. Ultrarush versus semirush initiation of insect venom immunotherapy: a randomized controlled trial. J Allergy Clin Immunol. 2012;130(1):162–8. 22460067 10.1016/j.jaci.2012.02.022

[22] Epstein TG, Liss GM, Berendts KM, AAAAI/ACAAI Subcutaneous Immunotherapy Surveillance Study. (2013–2017): Fatalities, infections, delayed reactions, and use of epinephrine autoinjectors. J Allergy Clin Immunol Pract. 2019;7(6): 1996–2003.e1. 10.1016/j.jaip.2019.01.058 30776526

[23] Phillips JF, Lockey RF, Fox RW, et al. Systemic reactions to subcutaneous allergen immunotherapy and the response to epinephrine. Allergy Asthma Proc. 2011;32(4):288–94. 10.2500/aap.2011.32.3446 21781404

[24] Golden DBK, Wang J, Waserman S, et al. Anaphylaxis: A 2023 practice parameter update. Ann Allergy Asthma Immunol. 2024;132(2):124–76. 38108678 10.1016/j.anai.2023.09.015

[25] Tunon-de-Lara JM, Villanueva P, Marcos M, Taytard A. ACE inhibitors and anaphylactoid reactions during venom immunotherapy. Lancet. 1992;340(8824):908. 1357311 10.1016/0140-6736(92)93314-d

